# Genetic Taster Status as a Mediator of Neural Activity and Swallowing Mechanics in Healthy Adults

**DOI:** 10.3389/fnins.2019.01328

**Published:** 2019-12-17

**Authors:** Angela M. Dietsch, Ross M. Westemeyer, William G. Pearson, Douglas H. Schultz

**Affiliations:** ^1^Department of Special Education and Communication Disorders, University of Nebraska–Lincoln, Lincoln, NE, United States; ^2^Center for Brain, Biology and Behavior, University of Nebraska–Lincoln, Lincoln, NE, United States; ^3^Department of Cellular Biology & Anatomy, Medical College of Georgia, Augusta University, Augusta, GA, United States

**Keywords:** swallowing, sensorimotor integration, taste, sensory perception, physiology, morphometry, genetic taster status, functional MRI

## Abstract

As part of a larger study examining relationships between taste properties and swallowing, we assessed the influence of genetic taster status (GTS) on measures of brain activity and swallowing physiology during taste stimulation in healthy men and women. Twenty-one participants underwent videofluoroscopic swallowing study (VFSS) and functional magnetic resonance imaging (fMRI) during trials of high-intensity taste stimuli. The precisely formulated mixtures included sour, sweet-sour, lemon, and orange taste profiles and unflavored controls. Swallowing physiology was characterized via computational analysis of swallowing mechanics plus other kinematic and temporal measures, all extracted from VFSS recordings. Whole-brain analysis of fMRI data assessed blood oxygen responses to neural activity associated with taste stimulation. Swallowing morphometry, kinematics, temporal measures, and neuroimaging analysis revealed differential responses by GTS. Supertasters exhibited increased amplitude of most pharyngeal movements, and decreased activity in the primary somatosensory cortex compared to nontasters and midtasters. These preliminary findings suggest baseline differences in swallowing physiology and the associated neural underpinnings associated with GTS. Given the potential implications for dysphagia risk and recovery patterns, GTS should be included as a relevant variable in future research regarding swallowing function and dysfunction.

## Introduction

Within the swallowing literature, the influence of taste stimulation on swallowing biomechanics has been an area of interest in healthy populations (Ding et al., 2003; Palmer et al., 2005; Pelletier and Dhanaraj, 2006; Leow et al., 2007; Wahab et al., 2011; Nagy et al., 2014b) and persons with swallowing impairments, or dysphagia (Pelletier and Lawless, 2003; Lee et al., 2012; Pauloski et al., 2012; Dietsch et al., 2019). Taste is particularly salient to swallowing, as gustation is important in eating and drinking behaviors and is mediated by multiple cranial nerves and neural structures that are integral in the swallowing response (Simon et al., 2006; Steele and Miller, 2010). Gustatory sensation is hypothesized to have a feed-forward effect on swallowing movement (Ding et al., 2003), as enhanced sensory stimulation may elicit faster and/or stronger swallowing responses. This theory is strengthened by studies reporting taste-related increases in neural activation in the cortical swallowing network (Babaei et al., 2010; Humbert and Joel, 2012); however, the evidence lacks consensus regarding taste’s effects on swallowing physiology and underlying neural activation.

Beneficial effects on the biomechanics of swallowing from a variety of taste stimuli in healthy people and persons with dysphagia include faster temporal parameters (Ding et al., 2003; Leow et al., 2007; Cola et al., 2010; Lee et al., 2012; Pauloski et al., 2012; Steele et al., 2012; Gatto et al., 2013) and more efficient/greater magnitudes of swallowing movements (Pelletier and Lawless, 2003; Palmer et al., 2005; Pelletier and Dhanaraj, 2006; Leow et al., 2007; Miura et al., 2009; Plonk et al., 2011; Wahab et al., 2011; Lee et al., 2012; Todd et al., 2012a, b; Nagy et al., 2014a, b; Pelletier and Steele, 2014; Dietsch et al., 2019). Although taste has shown positive effects on swallowing performance, these effects are not always statistically significant, may be conditional on certain taste stimuli, and/or may only be present at high concentrations of taste or with an interaction of multiple sensory inputs. Several studies report taste having negative or no effect on swallowing physiology in healthy adults (Butler et al., 2004; Hiss et al., 2004; Chee et al., 2005; Miyaoka et al., 2005, 2006) and persons with dysphagia (Hamdy et al., 2003) suggesting that positive results of taste on swallowing may be inconsistent for reasons that have not yet been elucidated.

Interpretation of the current literature is challenging for a multitude of reasons, which involve differences in (a) taste stimuli used, (b) measurement in swallowing outcomes, and (c) participant demographics. A paucity of evidence in the structural and functional neural representation of taste and swallowing also adds to the challenge in elucidating the relationship between these sensory inputs and motor outcomes. In addition to these issues, factors such as age, sex, and genetic predisposition create potentially significant sources of variability in modulating taste’s influence on brain activity and swallowing physiology (Bartoshuk et al., 1986, 1994; Bartoshuk, 2000; Kim et al., 2003).

Genetic taster status (GTS) is an inherited relative sensitivity to taste stimulation. It is assessed via chromosomal expression of the *TAS2R38* gene (Reed et al., 1999; Bartoshuk, 2000; Kim et al., 2003), density of the fungiform papillae on the tongue (Bartoshuk, 1993; Bartoshuk et al., 1994; Essick et al., 2003), and/or perceptual sensitivity to the bitter compound 6-n-Propylthiouracil (PROP; Bartoshuk, 1991; Smutzer et al., 2013). There are three broad classifications of GTS: nontasters, midtasters, and supertasters (Bartoshuk, 1991). Roughly half of the population are midtasters, one in four persons are supertasters, and women are more likely than men to be supertasters (Bartoshuk et al., 1994). GTS also influences perception of taste intensities, as supertasters report more intense reactions to taste stimuli in comparison to the other taster groups (Ko et al., 2000; Dietsch et al., 2014; Nagy et al., 2014a, b; Pelletier and Steele, 2014). This difference in oral perception could be a result of PROP tasters having higher amounts of gustatory papillae and taste pores (Bartoshuk et al., 1994; Bartoshuk, 2000; Essick et al., 2003) and thus experiencing increased sensory stimulation, and possibly a different combination of gustatory, chemosensory, and somatosensory input from oral stimuli, compared to nontasters (Karrer et al., 1992). These genetic, anatomical, and perceptual differences among GTS groups may manifest in different abilities and responses to taste stimuli in terms of both neural activation and swallowing movements.

A few studies have investigated GTS effects on neural activity using a range of neuroimaging designs and taste stimuli. Using magnetic resonance imaging (MRI), Bembich et al. (2010) reported significantly greater hemodynamic responses in areas of the prefrontal cortex (PFC) within supertasters after administration of PROP strips compared to nontasters, indicating the PFC’s active role in conscious processing of intense bitter tastes. Eldeghaidy et al. (2011) further investigated other brain regions that may be differentially activated based on GTS, and reported significant positive correlations between blood oxygen level-dependent (BOLD) responses and GTS in primary and secondary somatosensory cortices (SI, SII) the anterior cingulate cortex, and the anterior-, mid-, and posterior-insula after varied concentrations of isoviscous and isosweet fat emulsions. A functional near infrared spectroscopy (fNIRS) study by Mulheren et al. (2016) found no significant differences in hemodynamic activity among GTS groups in the sensorimotor cortices using sweet and sour stimuli. Similar to the neuroimaging literature, the evidence of an effect of GTS on swallowing biomechanics is also mixed. Supertasters have demonstrated stronger submental muscle activation (Pelletier and Steele, 2014), higher anterior lingual pressure generation (Nagy et al., 2014b; Pelletier and Steele, 2014), and longer swallow apnea durations (Plonk et al., 2011) in a variety of taste-intense stimuli compared with nontasters; however, other studies have reported GTS having no effects on these parameters (Todd et al., 2012a; Nagy et al., 2014a).

As part of a larger study, GTS was considered as a relevant factor in discerning the effects of taste stimulation on brain activity and swallowing physiology, as clarifying these relationships has important potential implications for dysphagia management. Specifically, the current study examines whether and how GTS influences neural hemodynamic responses and swallowing biomechanics within the same participants using standardized taste stimuli. It was hypothesized that general labeled magnitude scale (gLMS) scores, as an indicator of GTS, would be (H_1_) positively correlated to the magnitude of component swallowing movements and (H_2_) associated with differences in BOLD activity in taste- and swallowing-related neural areas.

## Materials and Methods

### Participants

This study included healthy adult volunteers recruited from the community via posters in local businesses and places of worship as well as in public buildings on the university campus. Volunteers were excluded from study participation if they had a history of neurological, taste, or swallowing disorders; injuries or surgeries to the orofacial region (aside from routine wisdom tooth extraction), or could not safely undergo MRI due to embedded metal or claustrophobia. The study protocol was approved by the primary investigator’s Institutional Review Board (#16762) and all participants provided written informed consent.

Twenty-one healthy adults (11 women, 10 men; mean age 27.66 years, range 19–49 years) participated in both data collection sessions. Distribution across sex and GTS is shown in Table 1 and is consistent with overall population distribution in that proportionately more women than men are supertasters, and more men than women are nontasters (Bartoshuk et al., 1994).

**TABLE 1:** Participant demographics.

**Group**	**Women**	**Men**	**Total *N***
Nontaster	2	7^∗^	9
Midtaster	4^∗^	2	6
Supertaster	5	1^∗∗^	6
Total *N*	11	10	21

*Twenty-one adult volunteers ranging 19–49 years of age (mean = 27.66) were distributed across sex and genetic taster status. ^∗^One participant from this group excluded from MRI analysis due to technical issues. ^∗∗^Excluded from CASM analysis as an extreme statistical outlier.*

### Stimuli

Five custom-mixed stimuli were prepared in distilled water for the functional magnetic resonance imaging (fMRI) trials and in a 40% weight/volume barium sulfate in distilled water mixture for the videofluoroscopic swallowing study (VFSS) trials. These included (1) intense sour, (2) sweet-sour, (3) lemon, (4) orange, and (5) unflavored (McBride and Johnson, 1987; Pelletier et al., 2004; Dietsch et al., 2019). All of the stimuli fell within the viscosity range for thin liquids according to the flow test methods and criteria recommended by the International Dysphagia Diet Standardization Initiative (Hanson et al., 2019). Table 2 delineates the composition of each tastant type.

**TABLE 2:** Taste stimuli.

**Tastant**	**Citric acid**	**Sucrose**	**Lemon extract**	**Orange extract**
Sour	2.7% wt/vol	N/A	N/A	N/A
Sweet-Sour	1.11% wt/vol	8% wt/vol	N/A	N/A
Lemon	1.11% wt/vol	8% wt/vol	1% vol/vol	N/A
Orange	1.11% wt/vol	8% wt/vol	N/A	1% vol/vol
Source	Fisher Scientific Citric Acid USP	C&H Granulated Pure Cane Sugar	McCormick Pure Lemon Extract	McCormick Pure Orange Extract

*All taste stimuli were precisely mixed using food- or pharmaceutical-grade materials. Distilled water served as the solvent for the neuroimaging trial stimuli. So the contrast would be visible on x-ray, a 40% weight/volume (wt/vol) barium sulfate (Fisher Scientific) suspension prepared in the lab served as the solvent for the videofluoroscopic swallowing trial stimuli.*

### Procedures

Participants underwent one session of data collection for VFSS, and another for MRI. These sessions were completed as proximately as scheduling allowed (mean 4.2 days, range 0–9 days). The MRI session was completed first for 16 participants and after the VFSS session for five participants.

**FIGURE 1:**
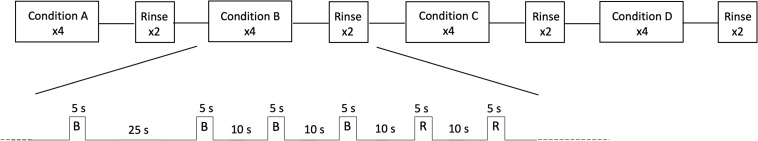
Study protocol. Participants completed four functional runs, each with a different sequence of the four taste stimuli (shown as conditions A–D, R = rinse) in a counterbalanced block design. The timeline for each run and a representative block are shown. Multiband volumes were collected every 1 s throughout the run.

Neuroimaging data were collected on a research-dedicated Siemens 3T MAGNETOM Skyra with 32-channel head coil. After positioning a participant in the scanner bed, investigators used medical tape to secure a short length of tubing (Skarda 1/8” OD clear food-grade urethane) to the participant’s lower face with the tip of the tube in the anterior portion of the participant’s oral cavity. This tubing was the endpoint for a custom-made stimulus dispensing system comprised of a series of modular pumps (Harvard Apparatus, Holliston, MA, United States) controlled by a PowerLab 16/35 (AD Instruments, Colorado Springs, CO, United States) and LabChart software (V. 8.1.13, AD Instruments, Colorado Springs, CO, United States). During functional image acquisition, TR pulses from the Siemens scanner were recorded within the LabChart software to enable precise registration of each volume to stimulus dispensation during analysis. Taste stimuli were administered in four counterbalanced blocks per functional run. Within each block, a single tastant was presented four times (3 ml per trial dispensed over 5 s with 15–30 s between onset of trials, see Figure 1), followed by two presentations of distilled water to rinse the oral cavity before starting the next tastant block. Four functional runs (430 volumes per run, gradient-echo T2^∗^-weighted imaging pulse sequence with GeneRalized Autocalibrating Partial Parallel Acquisition [GRAPPA] multi-band acceleration factor = 3, voxel size 2.5 mm^3^, field of view = 210 mm^2^, TR = 1 s, TE = 29.8 ms, flip angle = 60°, bandwidth = 2052 Hz/Px, echo spacing = 0.59 ms, interleaved) plus an anatomical T1 sequence (voxel size = 1 mm^3^, field of view = 256 mm^2^, phase encoding = anterior-posterior, TR = 2.20 s, TE = 3.37 ms, TI = 0.91 s, flip angle = 7°, bandwidth = 200 Hz/Px, echo spacing = 7.9 ms) were collected per participant as part of the larger study protocol.

Videofluoroscopic data were acquired at a local hospital by research personnel and cooperating radiology staff. Participants were seated with a lateral view that captured all relevant anatomical landmarks. Two trials of each stimulus were administered in a counterbalanced order via syringe at 45–55°F, for a total of ten 5-ml trials per participant. Immediately prior to each trial, the participant rinsed the oral cavity with tap water until no taste was discernable. Then, the syringe tip for the next trial was placed between the participant’s lips, emptied, and immediately withdrawn. Participants were prompted to swallow normally as soon as the syringe was removed from the oral cavity. Fluoroscopic swallowing images were captured at 30 pulses/s, and digitally recorded at 30 frames/s for further analysis.

In order to determine GTS, testing using a film impregnated with PROP (Sigma-Aldrich) was completed at the conclusion of their first data collection session. The researcher provided a simple explanation that taste perception is genetically influenced, and that the individual’s perception of the film could help us determine their genetic taste status. Then participants dissolved the PROP-impregnated strip on their tongue and then indicated the intensity of any associated taste on a gLMS (Smutzer et al., 2013). The intensity ratings were used by researchers to classify participants’ GTS during data analysis according to the criteria validated by Smutzer et al. (2013). Participants were not provided with information about the different GTS groups, how those groups typically perceive PROP, or their own GTS at any point during data collection.

### Analysis

#### VFSS Analysis

Each taste trial was clipped from the VFSS recording and coded such that researchers were blinded to stimulus type and GTS during data extraction. Oropharyngeal swallowing physiology of taste trials was assessed using a method called computational analysis of swallowing mechanics (CASM; May et al., 2017). CASM is the morphometric analysis of coordinate data mapping frame-by-frame displacement of anatomical landmarks. First, researchers who had achieved post-training interrater reliability of *r* ≥ 0.95 used a MATLAB-based semi-automated software tool to track twelve key anatomical landmarks frame by frame (Natarajan et al., 2015). Four of these anatomical landmarks (genial tubercle of mandible; posterior margin of hard palate; anterior tubercle of atlas; and the anterior inferior margin of C2) represent three relatively fixed levers within the skeletal structure surrounding the oropharyngeal swallow. They provide a reference framework for alignment of landmarks during the Procrustean fit portion of the analysis. Along with the anterior inferior margin of C4, they also are used to assess the relative posture of the cervical vertebrae during swallows. The remaining eight landmarks (anterior inferior margin of the hyoid; superior border of the upper esophageal sphincter (UES); anterior and posterior margins of the vocal folds; pit of the vallecula; and attachment of the superior and middle pharyngeal constrictors) represent the attachment points and foci of movement trajectory for muscle groups underlying pharyngeal swallowing mechanics (Hosseini et al., 2019). Considered together, these landmarks create a constellation representing overall pharyngeal shape changes during swallowing. The analysis process reveals whether the shape changes are associated with assigned grouping variables (such as GTS) as well as the magnitude and direction of coordinate shifts that contribute to the shape change. Ten percent of the study data were re-extracted to check for reliability; intra- and interrater correlation coefficients for CASM coordinate data were 0.933 and 0.975, respectively. Next, coordinates were compiled and imported into MorphoJ (Klingenberg, 2011). A Procrustes fit was performed to control for camera and subject position. An evaluation of the 3,607 sets of 12 coordinates representing pharyngeal stage swallowing against a multivariate distribution curve indicated that three sets of coordinates were found to be statistical outliers and excluded from analysis. A morphometric principal component analysis was performed to visually inspect the distribution of the sample. All data points from one subject, a male supertaster, lay outside of a 0.95 confidence interval of all other subjects and was therefore excluded from the analysis. To determine whether GTS impacted swallowing mechanics, a morphometric canonical variate analysis of coordinate data representing pharyngeal swallowing mechanics by GTS categories was performed in MorphoJ (Klingenberg, 2011). Ratings from PROP testing were used to stratify participants: nontasters = gLMS ≤ 20, midtasters = gLMS 21–40, supertasters = gLMS ≥ 41 (Smutzer et al., 2013). *Post hoc* discriminant function analysis yielded eigenvectors representing pairwise differences in pharyngeal phase mechanics by tastant. Eigenvector results were scaled using Mahalanobis distance in MorphoJ. A matrix transformation using MATLAB was performed on an exported eigenvector file (scaled vector graph) that aligns C1 and C4 vertebrae coordinates in order to visualize functional anatomical differences in swallow mechanics by tastant.

A second set of analyses of the VFSS images used more traditional kinematic and timing measures to further explore the effects of GTS on swallowing physiology. Pharyngeal constriction ratio (PCR; Leonard et al., 2006) was used to assess the magnitude of pharyngeal constriction by comparing pharyngeal volume at rest and at peak constriction. The pharyngeal phase duration was measured from the first frame of upward/forward movement of the hyoid to the frame in which the UES closes behind the bolus tail (Leonard et al., 2000). The UES distension was defined as the distance between the inferior and posterior opening of the UES when a majority of the bolus was passing through it. The parameters for each of these three measures were extracted from VFSS images and evaluated via a series of analyses of variance (ANOVA); significant alpha levels were set at 0.05 for the ANOVAs and Bonferroni adjusted to 0.0167 for *post hoc* testing.

#### MRI Preprocessing

Functional imaging reconstruction, processing, and analysis was conducted using Analysis of Functional Neuroimages (AFNI; Cox, 1996). Anatomical images were reconstructed and segmented using the standard FreeSurfer processing pipeline (Fischl et al., 2004; Desikan et al., 2006). Anatomical images were then non-linearly warped to MNI152_2009 template space using the AFNI program 3dQwarp, and the skull was removed. Echo-planar images for each run were despiked (spikes in each voxel’s time series are truncated), slice time corrected, aligned to the anatomical images and transformed to MNI space. Each volume was then registered to the volume with the minimum outlier fraction. Functional images were spatially smoothed using a 4 mm full-width at half maximum Gaussian filter, and skull stripped. The time course of each voxel was scaled to a mean of 100. We then ran a general linear model using the six motion estimates from volume registration as regressors of no interest. Additionally, we used up to third-order polynomials to model baseline and drift. Pairs of volumes where the Euclidean norm of the motion derivatives exceeded 0.4 were “scrubbed” and eliminated from further analysis. Finally, we modeled hemodynamic response functions using the “BLOCK” basis function at the onset time for each tastant as well as the rinse. The duration of the function was 6 s. Data from two participants (a female midtaster and a male nontaster) were excluded from further analysis due to technical issues with matching timelines of stimuli dispensation to image acquisition.

#### MRI Analysis

Beta values for rinse trials were subtracted from the beta value for each tastant. An ANOVA was conducted in AFNI using the 3dMVM program (Chen et al., 2014) with tastant as a within-subject factor, and GTS treated as a continuous between-subjects factor. A cluster-based approach was used to correct for multiple comparisons (Forman et al., 1995). We estimated the spatial smoothness of the residuals for each participant using a Gaussian plus mono-exponential function implemented with 3dFWHMx. The spatial autocorrelation function values were determined for each participant using the “-acf” option, and the mean values across participants (0.761, 2.956, 11.06) were calculated (Cox et al., 2017). Ten thousand random maps with these smoothness parameters were generated and thresholded at a voxel-wise *p* < 0.001. The largest surviving cluster from each of these simulations was recorded, and this distribution was used to estimate the probability of a false positive. Based on these estimations, we applied a cluster threshold to our data at a voxel-wise *p*-value of 0.001 and a minimum cluster size of ten contiguous voxels which resulted in a corrected two-tailed alpha of *p* < 0.05.

## Results

### Swallowing Physiology Outcomes

**FIGURE 2:**
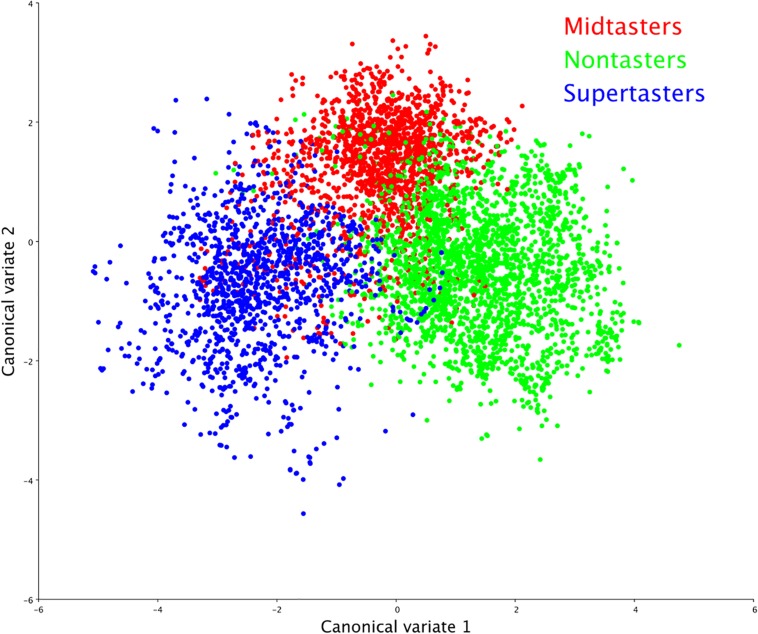
Swallowing mechanics by genetic taster status (GTS). Canonical variate analysis of swallowing physiology yielded distinct clusters by GTS group, indicating that taster status was a significant determinant (*p* < 0.0001) of swallowing shape changes.

Multivariate morphometric canonical variate analysis of the VFSS recordings by GTS showed statistically significant differences (*P* < 0.0001) in swallow mechanics across all comparisons (Figure 2) with Mahalanobis distances as follows: nontaster vs. midtaster (*D* = 2.40), midtaster vs. supertaster (*D* = 2.90), nontaster vs. supertaster (*D* = 3.44).

**FIGURE 3:**
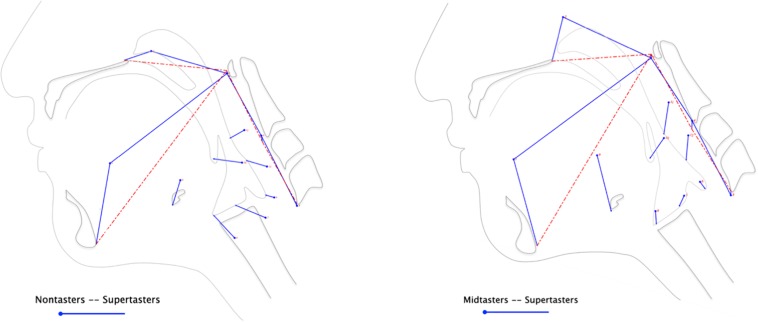
Differences in swallow mechanics by genetic taster status. Discriminant function analysis of the anatomical landmarks extracted from VFSS yielded eigenvectors illustrating the magnitude and direction of pharyngeal swallowing mechanics between groups. The mean variance of each landmark for nontasters **(left panel)** and midtasters **(right panel)** is represented by the circle origins of the eigenvectors, whereas the supertasters’ landmark variances are depicted by the endpoint of each eigenvector.

In the subsequent discriminant function analysis, eigenvectors illustrate the differences in supertaster pharyngeal swallowing mechanics compared to those of nontasters (Figure 3, left panel) and midtasters (Figure 3, right panel). As depicted by the length and direction of the eigenvectors, supertasters demonstrated greater pharyngeal constriction and increased head and neck flexion than either of the other groups. Additionally, the CASM results indicated increased laryngeal elevation, anterior hyoid excursion, and pharyngeal shortening in supertasters as compared to nontasters.

The three kinematic and timing variables analyzed via one-way ANOVAs further characterized the impact of taster status group on swallowing physiology (Table 3). Consistent with the results from the CASM, supertasters had significantly smaller PCR values than midtasters and nontasters, indicating they had the largest average magnitude of pharyngeal constriction across taster groups. Additionally, supertasters had the shortest pharyngeal phase duration of swallowing; it was significantly shorter than for midtasters but not nontasters. Although there were no significant mean differences of GTS and UES distension, supertasters had the largest value of UES distension (*M* = 23.208, *SD* = 8.126) with midtasters (*M* = 20.389, *SD* = 4.684) and nontasters (*M* = 21.285, *SD* = 5.507) having similar UES distension values.

**TABLE 3:** Analyses of variance for kinematic and timing variables across genetic taster groups.

**Variable**	***df***	***F***	***p***	**Effect description**
Pharyngeal constriction ratio (PCR)	2, 182	5.191	0.006	^∗^Supertasters < Midtasters ^∗^Supertasters < Nontasters Midtasters = Nontasters
Pharyngeal phase duration (PPD)	2, 180	9.305	<0.001	^∗^Supertasters < Midtasters Supertasters = Nontasters Midtasters > Nontasters
UES distension	2, 190	2.991	0.053	N/A

**Post hoc* pairwise comparisons for significant main effects revealed that supertasters exhibited more efficient swallows (lower PCR and PPD) than mid- and nontasters. ^∗^Denotes a statistically significant relationship at Bonferroni-adjusted alpha levels of 0.0167.*

### MR Outcomes

**FIGURE 4:**
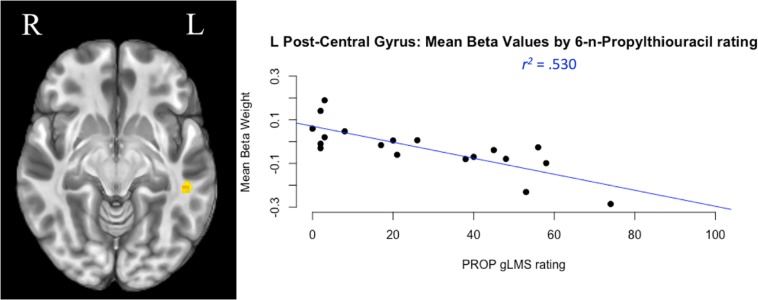
Taste perception and cortical activity. The largest cluster of neural activation during taste stimulation compared to plain water was located in the left post-central gyrus, the somatosensory cortex **(left panel)**. A regression analysis compared participants’ genetic taste sensitivity [measured by general labeled magnitude scale (gLMS) ratings of taste intensity in response to 6-n-Propylthiouracil (PROP) stimulation] to their change in neural activity (measured by mean beta weight of BOLD signal). Results reflect that during taste stimulation trials, persons with greater taste sensitivity (a higher PROP gLMS rating) exhibited lower levels of BOLD activation in this region compared to person with lower PROP ratings **(right panel)**.

The whole-brain neuroimaging analysis identified regions where BOLD activity varied as a function of GTS as measured by gLMS intensity ratings for the PROP strip. The analysis revealed two clusters of BOLD signal associated with main effects of GTS. Their coordinates, cluster size, and effect descriptions are summarized in Table 4. The smaller cluster was located in the superior temporal gyrus (STG), a region implicated in a broad range of functions including swallowing (Martin et al., 2004; Shibamoto et al., 2007; Peck et al., 2010). The larger cluster was located in the left post-central gyrus, specifically in a portion of S1 associated with orofacial sensation (Haggard and de Boer, 2014). Within this area of S1, participants’ gLMS intensity rating for the PROP strip testing (a reflection of GTS) accounted for 53% of the variance in hemodynamic response in this key region (*F*[1,17] = 19.17, *p* < 0.0001). As depicted in Figure 4 (right panel), higher PROP gLMS ratings, reflecting greater sensitivity to taste, were associated with lower BOLD activation during trials that involved tastant presentations as compared to during the control condition.

**TABLE 4:** Main effects of genetic taster status.

**Cluster**	**Coordinates (MNI152-2009)**			**Volume (mm^3^)**	***F*(1,17)**	***R*^2^**	**Effect description**
	**RL**	**AP**	**IS**				
Post-central gyrus	–50	–32	51	203	19.17	0.530	Higher gLMS → more negative beta weight
Superior temporal gyrus	26	5	–32	156	21.57	0.560	Higher gLMS → more negative beta weight

*Two clusters survived whole brain analysis and cluster-based correction for multiple comparisons. Higher scores on the general labeled magnitude scale (gLMS) during 6-n-Propylthiouracil testing were associated with supertaster status. RL = right/left, AP = anterior/posterior, IS = inferior/superior.*

## Discussion

The present analysis assessed the relationships between GTS, swallowing physiology, and neural activity in healthy adults in response to precisely mixed taste stimuli. The results supported our hypotheses, with supertasters exhibiting greater amplitudes of critical swallowing movements (H_1_) and a different pattern of neural activation (H_2_) compared to mid- and nontasters.

Analysis of swallowing physiology via CASM and other kinematic/timing variables confirmed previous reports of GTS-dependent differences in swallowing movements. Overall, supertaster status was associated with greater magnitude of swallowing movements compared to mid- and nontasters, as evidenced by CASM’s discriminant function analysis eigenvectors and the kinematic pharyngeal constriction findings. In addition to the movement parameters, supertasters had the shortest pharyngeal swallow phase duration (the time required to move the bolus through the pharynx), suggesting more efficient swallowing physiology than both mid- and nontasters. This may seem counterintuitive initially, and some studies have reported increased pharyngeal constriction to be associated with longer contraction of the relevant musculature (Inamoto et al., 2018; Molfenter et al., 2018). However, these findings involved within-subject comparisons of swallowing dynamics across varying conditions or cues that yielded different levels of effort in the swallows, i.e., the same basic motor plan executed to varying endpoints. In contrast, Steele et al. (2019) reported that whereas pharyngeal constriction ratios were significantly different during normal-effort swallows of varying bolus consistencies, healthy swallowers exhibited less than 20% variability in the timing parameters of those swallows. In the present study, we report across-subject comparisons, which may have introduced variability in the speed and acceleration of component movements from one person’s motor plan to another’s. This would confound the stability of the larger amplitude/longer duration relationship. Additionally, our measure of duration was not based on timing of component movements or muscle contractions, but on how fast the bolus completed the pharyngeal phase (spanning from the first frame of hyolaryngeal excursion to when the UES closes behind the bolus tail; Leonard et al., 2000). So even though the duration of pharyngeal movements/contractions may be longer for those exhibiting greater magnitudes of constriction (supertasters), that is not directly tied to the speed with which they moved the bolus. Although we did not measure speed or acceleration of component movements, perhaps the baseline swallowing mechanics of the supertasters included greater speed and magnitude of pharyngeal constriction, leading to faster propulsion of the bolus through the pharynx and into the UES.

Neuroimaging also delineated GTS-linked differences, though the reduced activation in S1 and STG for supertasters as compared to mid- and nontasters may seem counterintuitive; one might expect the increased sensory input inherent to supertasters to be associated with increased S1 and STG activation. Broader consideration of the extant literature in the fields of cognitive psychology, sensory processing, and neuroscience, however, offers several possible explanations for these findings.

Researchers in cognitive psychology, for example, note that more intelligent individuals exhibit smaller activation amplitude while performing cognitive tasks than lower intelligence individuals (Haier et al., 1992; Neubauer and Fink, 2009). This finding has been referred to as the Neural Efficiency Hypothesis. Further evidence of this hypothesis has suggested that it is sensitive to other variables such as task demands (Dunst et al., 2014), and gender (Lipp et al., 2012). In addition to more efficient brain activation patterns, other studies have suggested that this efficiency effect may also be apparent in updates to brain network communication (Schultz and Cole, 2016; Zuo et al., 2018). If applied to taste stimulation, this neural efficiency phenomenon could suggest that by virtue of their higher density of taste receptors, supertasters are relatively more adept at processing taste-related sensation and thus need to devote fewer neural resources than mid- and nontasters, or that their neural patterns for processing the gustatory, chemosensory, and somatosensory inputs associated with taste are simply different than their counterparts. Prior studies have identified increased activity in the STG during swallows at higher effort levels (Peck et al., 2010) and of more complex boluses (Shibamoto et al., 2007). The lower STG activations in supertasters, combined with their generation of greater movement magnitudes more quickly than their mid- and nontaster counterparts in this study, suggest that perhaps supertasters are more efficient at motor aspects of swallowing as well.

The sensory processing literature offers other possible explanations for the lower S1 and STG activations in supertasters. Abundant evidence supports that S1 has a critical role in pain perception (Bushnell et al., 1999), and undergoes neuroplastic changes in response to pain (see Kim et al., 2017 scoping review). For example, persons with acute low back pain exhibited smaller activations in the sensorimotor cortex than pain-free peers in response to non-noxious afferent inputs (Chang et al., 2019), suggesting that decreased S1 activations may be a rapidly developing compensatory response to discomfort. Similarly, individuals with chronic pain disorders exhibited reduced resting state activity in STG (Zhao et al., 2017). Perhaps supertasters’ neural systems perceive intense taste-related stimulation, which may include somatosensory and chemesthetic (and possibly nocioceptive for certain stimuli such as capsaicin or intense sour) components, as uncomfortable or noxious. To compensate, supertasters’ neural networks might downregulate S1 and STG activity in response to taste stimulation in the same way that persons with pain downregulate other tactile stimuli. Additionally, it is well-established that pain perception and S1 activation are also modulated by attentional factors (Zeidan et al., 2011) and enhancement of other sensory modalities (Sharma et al., 2016). Under this principle, supertasters’ inherent sensitivity to taste stimuli could yield a neural network that de-prioritizes somatosensory/chemesthetic information in favor of gustatory inputs, resulting in comparatively less S1 activity than mid- and nontasters.

A third consideration involves the extant neuroscience literature regarding taste-related sensation, or rather the gaps that exist in our current understanding of the complex underlying networks. Investigators using various stimuli and neuroimaging technologies have identified a range of brain regions and timing patterns that appear to be involved in the processing of taste stimuli generally (Babaei et al., 2010; Humbert and Joel, 2012), and specifically with regard to GTS (Bembich et al., 2010; Eldeghaidy et al., 2011; Mulheren et al., 2016). While all of these contributions are valuable, a clear picture of the neural pathways for processing of the multimodal inputs associated with taste stimulation, much less one that accounts specifically for GTS, has yet to emerge. Another study reporting GTS-related differences in S1 activation showed opposite effects to ours, with greater activation in S1 for supertasters (Eldeghaidy et al., 2011). However, the trials of fat emulsions used in that work have different mouth-coating and dispersive qualities than our taste stimuli (Drewnowski, 1992). In addition to contrasting taste stimuli, different prioritization of gustatory, chemosensory, and somatosensory inputs by supertasters could contribute to differing somatosensory-specific S1 activation patterns.

The baseline differences in swallowing physiology and neural activation in healthy persons demonstrated by these results may help explain the disparate results in previous studies of swallowing function. If people have genetic differences in their baseline swallowing movements and responses to oral sensation, GTS could be a confounding variable in capturing outcomes of enhanced sensory stimulation on swallowing, or in responding to particular sensorimotor intervention strategies. Additionally, symptoms of dysphagia could manifest differently even in persons with similar neurological or structural insults due to variations in baseline swallowing movements and neural networks associated with their GTS. If so, GTS could influence the severity and prognosis of dysphagia, and therefore may be an important variable to consider in dysphagia assessment and management. Further investigation is necessary to determine whether the incidence of dysphagia is different across GTS groups, and whether there are fundamental differences in the functional and anatomical neural connections that could influence one’s susceptibility to taste- and swallowing-related impairments and responsiveness to specific interventions.

The present results support that supertasters require less S1 activation and similar M1 activation in order to achieve greater amplitude and efficiency of swallowing movements. In other words, supertasters may be neurologically predisposed to produce more optimized swallows at baseline compared to non- and midtasters. While this raises intriguing questions about the role of GTS in the risk of developing dysphagia as well as the potential to recover from it, additional work is necessary to address limitations of the current work and further examine the underlying mechanisms. For example, CASM comparisons would benefit from an increased sample size that would enable stratification by sex to account for morphological differences that may affect functional anatomy as represented by the eigenvectors. This may improve the precision of vectors indicating how the multiple elements of pharyngeal swallowing mechanics are impacted by GTS. Larger sample size could also increase the statistical power to detect additional shifts and/or clusters of BOLD signal change in other brain regions. Also, the incorporation of genetic testing in future work will allow for more precise assessment of taster status haplotype than is possible with the PROP testing utilized here. Additionally, the tastants used here were developed to mirror those that elicited the most efficacious swallowing mechanics in previous studies, but it is possible that other tastant types may have similar or even better effects. Although other studies reported no differences in measures of taste perception and swallowing physiology for the barium and non-barium versions of sweet, sour, and unflavored taste stimuli similar to the formulas used here (Dietsch et al., 2014; Nagy et al., 2014a), the stimuli in the fMRI and VFSS are not identical because of their barium status, which may have impacted the respective results in unappreciated ways. Likewise, the fact that some participants underwent VFSS first whereas others completed the MRI first could have confounded results. Finally, the current work focuses on swallowing mechanics and neural activity in healthy persons, so further work is necessary to assess whether these relationships hold in persons with dysphagia. Nonetheless, these findings raise questions about whether supertasters may also require less neural recruitment to adapt the swallowing motor plan based on the intraoral stimulus being swallowed, which may have significant implications for treatment selection for individuals with dysphagia.

In summary, the current study offers a unique contribution to the extant literature by using GTS as a covariate in both neuroimaging and swallowing physiology data from the same participants using standardized taste stimuli. Comparison of swallowing mechanics revealed increased amplitude and efficiency of swallowing physiology in supertasters compared to mid- and nontasters. Further, S1 activation inversely correlated to PROP rating, such that supertasters appear to devote fewer neural resources to somatosensory aspects of oral stimulation than mid/nontasters. The influence of GTS on swallowing biomechanics and neural substrates is a compelling new finding, and these preliminary results suggest that GTS may be a relevant consideration in future swallowing-related research.

## Data Availability Statement

The raw data supporting the conclusions of this article will be made available by the authors, without undue reservation, to any qualified researcher who makes reasonable and scientifically feasible requests. Data sharing will occur after appropriate institutional data use agreements have been completed.

## Ethics Statement

This study was conducted under the guidance and approval of the Institutional Review Board of the University of Nebraska–Lincoln, with written informed consent from all participants in accordance with the Declaration of Helsinki.

## Author Contributions

AD conceptualized the study, obtained funding, designed the study, constructed the requisite equipment, recruited the participants, collected the data, oversaw all the analyses, and wrote the manuscript. RW helped to recruit participants, collect data, and analyze data, and wrote portions of the manuscript. WP designed and conducted the morphometric analysis. DS designed and conducted the neuroimaging analysis, and wrote portions of the manuscript. All authors contributed to the manuscript editing, revision, read, and approved the submitted version.

## Conflict of Interest

The authors declare that the research was conducted in the absence of any commercial or financial relationships that could be construed as a potential conflict of interest.
